# Unmasking programmed cell death in rhinovirus-driven asthma pathology

**DOI:** 10.1042/BST20250090

**Published:** 2026-09-24

**Authors:** Wayne Cawthorne, Youming Zhang, Nicole Val, Michelle Nelson, Anna Raevski, Kazuhiro Ito, Reena Ghildyal

**Affiliations:** 1 Walter and Eliza Hall Institute of Medical Research, Melbourne, Australia; 2 Department of Medical Biology, University of Melbourne, Melbourne, Australia; 3 National Heart and Lung Institute, Imperial College, London, U.K.; 4 Faculty of Science and Technology, University of Canberra, Canberra, Australia

**Keywords:** asthma, asthma exacerbations, programmed cell death, rhinovirus

## Abstract

Rhinovirus (RV) infection is responsible for the majority of cases of viral exacerbations of asthma. Understanding the cellular and molecular mechanisms that cause asthma exacerbations is an absolute requirement for the development of effective treatments. Emerging literature suggests that cell death may have an important role in RV-induced exacerbations. Several types of programmed cell death (PCD) have been implicated in asthma, including apoptosis, necroptosis, pyroptosis, and ferroptosis. Increased presence of apoptotic airway epithelial cells in the sputum of adult and paediatric asthma patients is suggestive of defects in apoptosis. Elevated levels of phosphorylated mixed lineage kinase domain-like protein and receptor-interacting serine/threonine-protein kinase 3, markers of active necroptosis, have been found in the serum of asthmatic patients. Gasdermin B, a member of the gasdermin family of pyroptosis executioner proteins, has a strong genetic linkage to severe asthma. Expression level of GSDMB correlated with asthma exacerbations and antiviral pathways. Lipid peroxidation and increased airway iron levels, both suggestive of ferroptosis, have been observed in asthma patients. RV infection of epithelial cells triggers an inflammatory response, which can provide the necessary pro-inflammatory chemokines and cytokines to initiate PCD, such as tumour necrosis factor-α. RV exploits PCD such as apoptosis, necroptosis, pyroptosis, and ferroptosis to aid its replication and release, while modulating them probably to limit immune response. In the present review, we investigate the concept that pro-inflammatory PCD pathways modulated by RV infection in the context of asthma lead to prolonged inflammatory and immune responses that are key to asthma exacerbations.

Asthma is a chronic inflammatory pulmonary disease of the conducting airways, usually caused by an immunological reaction. Asthma causes episodes of bronchoconstriction, which results in coughing, wheezing, breathlessness, and tightness of the chest. These symptoms result from increased sensitivity of the airways, bronchial wall inflammation, and increased mucus secretions [1]. Asthmatic bronchoconstriction can be triggered in response to a variety of stimuli, with the main causes being viral respiratory infections and exposure to offending allergens [2]. Rhinovirus (RV) infection is the major cause of virus-induced asthmatic attacks in adults and children [3]. When compared with non-asthmatics, there are more severe lower respiratory tract symptoms observed in asthmatics who take longer to clear the virus from their lungs [4–6].

Several different programmed cell death (PCD) pathways, including apoptosis, necroptosis, pyroptosis, ferroptosis, and autophagy, have been implicated in the pathogenesis and regulation of asthma. Interestingly, RV infection has also been linked to modulation of apoptosis, autophagy, and necroptosis [7–9]. The question then arises—is RV modulation of PCD, overlaid on already dysregulated pathways, critical to the observed asthma exacerbation? Which leads to the corollary: are specific PCD pathways viable targets for therapeutic intervention?

In the present review, we bring together the literature linking asthma and RV infection to PCD pathways to address the above questions and direct future research for therapeutic development.

## Programmed cell death pathways

We begin the review with an overview of the most relevant PCDs that have been implicated in asthma and in RV infection. We have decided not to include autophagy in the present review. Autophagy, although often reviewed with PCDs, is essentially a cyclic cleanup and repair mechanism where death is not the outcome. PCD provides a mechanism for the removal of non-functioning cells. PCD pathways are highly complex and tightly regulated to ensure healthy cells are not destroyed and mutant or diseased cells do not undergo mitotic growth. Extensive crosstalk and co-regulation exist, and recently identified PANoptosis suggests co-ordinated activation across PCD pathways [10].

### Apoptosis

Apoptosis is characterised by cell shrinkage, cell membrane blebbing, nuclear condensation, and cell fragmentation [11]. Apoptosis is activated via two main pathways [12] (Figure 1, shown in blue).

**Figure 1:**
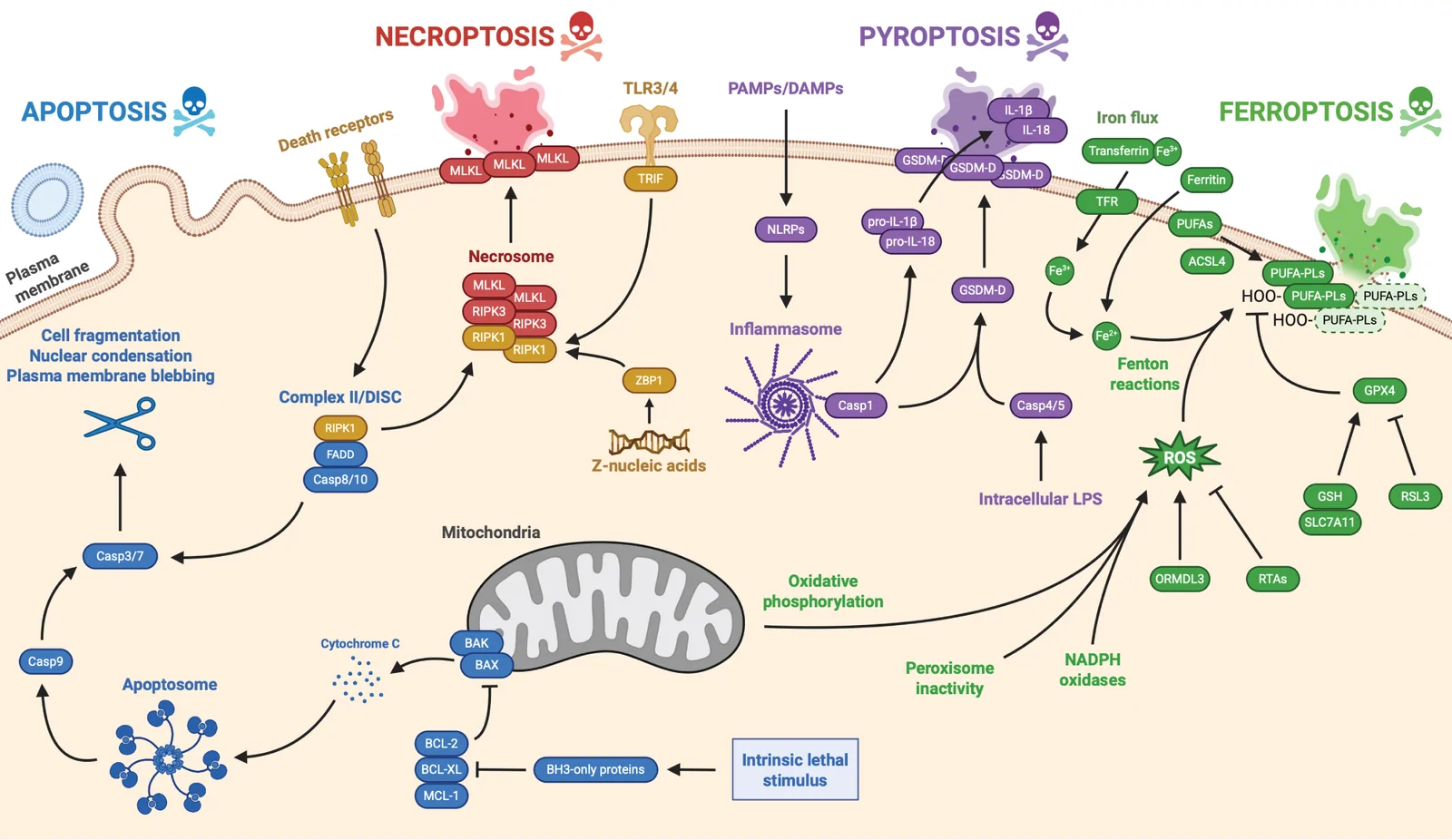
PCD pathways relevant to RV-induced asthma exacerbations Apoptosis (shown in blue) can be initiated through the intrinsic or extrinsic pathway. Intrinsic stimuli lead to the up-regulation of pro-apoptotic BH3-only proteins that inhibit anti-apoptotic Bcl-2 family proteins to allow BAX/BAK pore formation in the mitochondria. This releases cytochrome C into the cytoplasm, inducing apoptosome formation, which results in caspase-9 activating caspase-3/-7 to cause cell fragmentation, nuclear condensation, and plasma membrane blebbing to drive apoptotic cell death. The extrinsic apoptotic pathway occurs downstream of death receptors (DRs) in particular cell contexts, resulting in Complex II/DISC-driven and Casapse-8/-10-dependent activation of Caspase-3/-7 to drive apoptosis. Necroptotic cell death (shown in dark red) occurs after necrosome formation by DRs via RIPK1, TLR-3/4 via TRIF, and Z-nucleic acids via Z-DNA binding protein (ZBP)1, which activates mixed lineage kinase domain-like protein (MLKL) and causes plasma membrane rupture and cell lysis. The canonical pyroptotic pathway (shown in purple) eventuates when pathogen-associated molecular patterns (PAMPs)/damage-associated molecular patterns (DAMPs) induce inflammasome formation, such as via NLRPs, which results in Caspase-1-dependent GSDMD activation and pore formation and the extracellular release of active IL-1β and IL-18. Non-canonical pyroptosis can eventuate via intracellular lipopolysaccharide (LPS), causing Caspase-4/-5 and downstream GSDMD activation. Ferroptosis (shown in green) can eventuate when increases in reactive oxygen species (ROS) via several sources undergo Fenton reactions with intracellular iron to cause lipid peroxidation, leading to plasma membrane damage and cell lysis.

Triggered by disruptions in cellular homeostasis, intrinsic apoptosis occurs via changes to the mitochondrial outer membrane permeability, leading to the release of pro-apoptotic factors. These factors activate the caspase-dependent mitochondrial pathway of apoptosis [11–13]. Intrinsic apoptosis is controlled and regulated by the B-cell lymphoma 2 family proteins through pro- and anti-apoptotic functions (reviewed in [14]). Extrinsic apoptosis relies on the activation of death receptors (DRs) to initiate and induce cell death. Receptors such as the Fas cell-surface DR or the tumour necrosis factor receptor 1 are activated by binding with their ligands (FasL and TNF-α, respectively), triggering intracellular signalling pathways that result in the activation of caspase-8 [12,13]. Both apoptosis pathways culminate with activation of executioner caspases (e.g., caspase-3/-7), which cleave a variety of substrates, resulting in the typical morphological and biochemical changes seen in apoptosis [12].

### Necroptosis

Necroptosis is a programmed variant of necrosis, which can provide an alternative suicide mechanism in cases of apoptosis malfunction or inhibition [15,16]. Necroptosis evolved as an anti-pathogen defence mechanism, and many pathogens encode proteins that inhibit necroptosis [17,18]. In contrast with apoptosis, necroptosis results in cell swelling and membrane blowout [19] (Figure 1, shown in dark red). Several stimuli can induce necroptosis, including cytokines such as TNF and pathogen components such as lipopolysaccharide and Z-RNA [20].

Necroptosis is terminally executed by the plasma membrane permeabilising protein, MLKL [21,22], which, once activated, causes lethal necroptotic cell death. MLKL’s cytotoxic potential is dependent upon its phosphorylation by receptor-interacting serine/threonine-protein kinase 3 (RIPK3) within a ∼2 megadalton signalling complex termed the necrosome [20,23]. Formation of the necrosome is dependent upon RIPK3’s oligomerisation by RIP homotypic interaction motif-domain-containing proteins RIPK1, Toll/interleukin-1 receptor domain-containing adaptor protein-inducing interferon (IFN) β (TRIF), and ZBP1 [20].

Necroptosis causes inflammation through the production and release of DAMPs upon plasma membrane rupture. DAMPs recruit pro-inflammatory cytokines and chemokines to the cell area, in turn activating and attracting immune cells [24].

### Pyroptosis

Pyroptosis is activated in response to disturbances in extra- or intracellular homeostasis and is dependent on formation of gasdermin (GSDM) pores [13]. Increasing evidence suggests pyroptosis involvement in respiratory diseases, including asthma and chronic obstructive pulmonary disease [25,26].

Most GSDMs contain an N-terminal pore-forming domain (NT) and a C-terminal inhibitory domain (CT) connected by a linker [27,28]. Cleavage within the linker releases the NT from inhibition. The NT translocates to the plasma membrane, binds to negatively charged lipids, undergoes conformational changes, and oligomerises, forming a pore [29,30]. Influx of extracellular water causes cell swelling and osmotic lysis with release of intracellular content, including lactate dehydrogenase (LDH) [31] and high mobility group box-1 [32].

The canonical pathway of pyroptosis activation begins with cytosolic inflammasome sensors that oligomerize upon activation and bind to an adaptor protein, apoptosis-associated speck-like protein (ASC, Figure 1, shown in purple). NLR family pyrin domain-containing (NLRP) 3 is the most studied sensor, activated in response to many different PAMPs and DAMPs [33–36]. NLRP1, a related sensor, has been implicated in infection and can be stimulated by dsDNA [37], dsRNA [38], and reduction in cellular ATP [39]. ASC links to pro-caspase-1, which undergoes self-cleavage to form active caspase-1. Caspase-1 cleaves GSDMD, releasing active GSDMD-NT [28,40].

Several isoforms of Gasdermin B (GSDMB) have been described, with some being pyroptotic on cleavage by granzyme A, while others are unable to form functional pores [41,42]. The CT domain of GSDMB lacks auto-inhibitory function, allowing full-length GSDMB to bind to membranes without pore-forming activity [43].

### Ferroptosis

Ferroptosis is involved in many diseases from cancer to autoimmune diseases. Ferroptosis is a form of non-apoptotic cell death driven by iron-dependent lipid peroxidation [44], described in 2012, following studies showing that death of mammalian cells deprived of extracellular cystine coincides with loss of glutathione [45,46]. The generation of ROS and subsequent hydroxyl radical (OH)-mediated lipid peroxidation culminating in the accumulation of phospholipid peroxides and plasma membrane damage are the core events in ferroptosis [47] (Figure 1, shown in green).

Iron is imported by extracellular transferrin that binds the transferrin receptor on the cell membrane and exported out by ferroprotein [48]. Accumulation of iron can drive the Fenton reaction to propagate lipid peroxidation, thereby increasing oxidative damage. Cystine transporter solute carrier family 7 member 11 and glutathione peroxidase (GPX) 4 are the key players in antioxidant defence against ferroptosis. GPX4 reduces complex hydroperoxides to their corresponding alcohols, thereby interrupting the lipid peroxidation chain reaction [49,50]. RAS-selective lethal 3 inhibits the activity of GPX4, thus inducing ferroptosis [51].

## Programmed cell death in RV infection

RV modulates PCD pathways to evade the host immune response and maybe to mediate viral release from cells. Apoptosis and autophagy have been proposed as mechanisms for RV release, while recent work implicates necroptosis in RV infection [7–9,52]. Accumulating literature indicates that RV components interact with, interrupt, or modulate several cell-signalling cascades in the infected cell to support the virus lifecycle [15,53–55]. The various published direct and indirect effects of RV proteins on PCD pathways are depicted in Figure 2 (in bright red).

**Figure 2:**
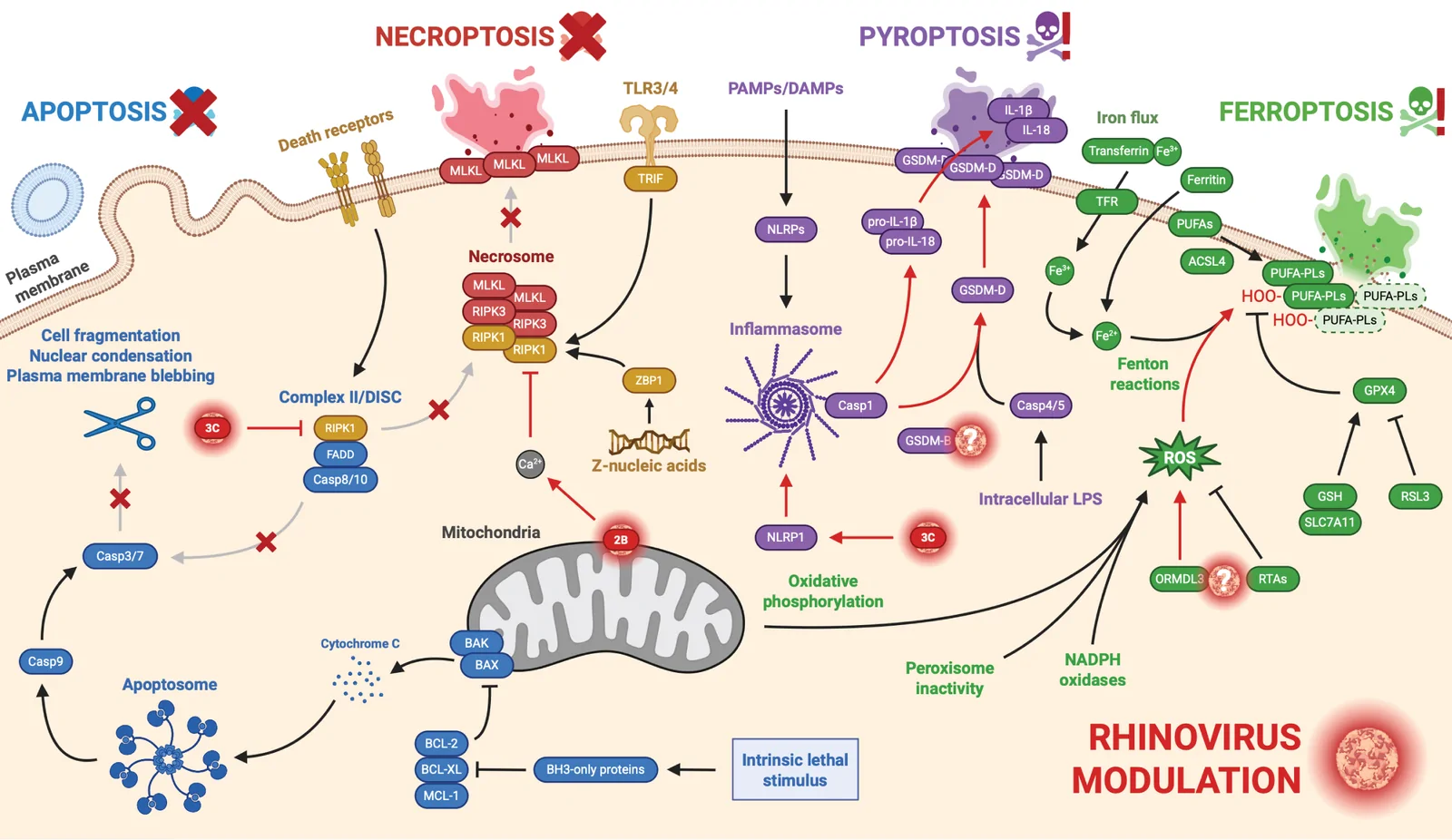
RV modulation of PCD pathways RV infection in cells leads to the release and activation of viral effector proteins that modulate PCD pathways. 3C protease cleaves and inactivates RIPK1 to prevent Complex II/DISC and necrosome activation, resulting in inhibition of extrinsic apoptosis and necroptosis pathways. 3C protease also cleaves and activates NLRP1, which drives inflammasome activation and pyroptosis signalling. 2B inserts into the mitochondria and causes Ca^2+^ leakage, which can modulate apoptosis/necroptosis signalling during RV infection. The gene *GSDMB* encoding the pro-pyroptotic protein GSDMB is associated with RV-associated disease exacerbations. The gene *ORMDL3* encoding the pro-ferroptotic protein ORMDL3 is associated with RV-associated disease exacerbations and may have multiple pathways of influencing RV infection. Direct and indirect effects of RV infection are shown in bright red colour.

### Apoptosis

Picornaviruses modulate apoptosis at various stages of their lifecycle [56–62]. Typical morphological features of apoptosis are observed following RV14 infection of HeLa and bronchial epithelial cells (BECs), corresponding with high-molecular-weight DNA fragmentation, activation of caspase-9 and caspase-3, poly (ADP-ribose) polymerase cleavage, and cytochrome c translocation, all of which are hallmarks of intrinsic apoptosis [52]. RV infection of HeLa cells has also been shown to cleave pro-caspase-8, a key step in the extrinsic apoptosis pathway [63].

Early induction of apoptosis in virus-infected cells is critical to the innate antiviral response, preventing establishment and transmission of viral infection (reviewed in [64,65]). Inhibition of apoptosis in RV-infected BECs results in enhanced virus release [66].

RV proteases 2Apro, 3Cpro, and its precursor 3CD have been shown to cleave several nucleoporins, disrupting nucleocytoplasmic transport and potentially inhibiting apoptosis through the mis-localisation of caspases [66–73]. RV proteases also cleave proapoptotic adaptor proteins, resulting in the down-regulation of apoptosis. RV 3Cpro-mediated cleavage of RIPK1 modulates the induction of extrinsic apoptosis. RIPK1 cleavage by caspase-8 is a key step in the extrinsic pathway, committing the cell to apoptotic cell death. Subsequent cleavage of RIPK1 by 3Cpro following RV16 infection in HeLa cells halts the progression of apoptosis [7,9], a finding consistent for RV-A and -B species, major and minor groups, suggesting that 3Cpro cleavage of RIPK1 to halt apoptotic progression is a trait conserved across most, if not all, RV strains [74].

Sudden release of calcium ions from the ER can initiate intrinsic apoptosis. Following infection, RV 2B protein inserts into intracellular membranes, allowing a slow release of calcium ions into the cytoplasm [75], resulting in inhibition of apoptosis via this pathway [75,76].

### Necroptosis

Recent investigations have identified that 3Cpro cleavage of RIPK1 inhibits apoptosis progression, resulting in the induction of caspase-independent cell death with necrotic characteristics, such as necroptosis [7,9].

RV infection mediates the release of various cytokines and chemokines [77–80]. Among these, pro-inflammatory proteins interleukin (IL)-1 and TNF-α are released in response to RV infection and have been shown to induce necroptosis [81,82]. This may suggest that necroptosis is mediated through RV-induced inflammation; however, there is no definite supporting data.

RV infection has been associated with increased levels of cytoplasmic calcium, probably resulting from RV protein 2B and associated calcium store disruption [75]. Increased cytosolic calcium in human neuroblastoma cells resulted in the inhibition of caspase-8, calcium/calmodulin-dependent kinase II phosphorylation, and the induction of necroptosis [83].

### Pyroptosis

RV-induced inflammasome activation leading to IL-1β secretion and caspase-1 cleavage has been described [84]. This process was associated with NLRP3 and driven by cytosolic calcium accumulation upon activity of RV protein 2B. Interestingly, RV 3Cpro was shown to cleave NLRP1, leading to the latter’s activation. Cleavage immediately after the pyrin domain opens an N-terminal glycine, which recruits cellular cullin^ZER1/ZYG11B^ machinery (the Gly/N-degron pathway of the ubiquitin-proteasome degradative system), leading to degradation of the inhibitory NLRP1 N-terminal fragment and release of active UPA-CARD segments [85]. Thus, RV-dependent NLRP1 activation causes inflammasome and caspase-1 activation, processing, and release of IL-18 and IL-1β as well as GSDMD cleavage in BECs.

3Cpro from other picornaviruses, including enterovirus (EV) 71 and 68, also cleaves and activates human NLRP1 [86]. EV71 3Cpro down-regulates pyroptosis by direct cleavage of GSDMD at glutamine at position 193 (Q193), which is outside the linker domain [87], resulting in a pro-viral effect. Activation of NLRP3 by the EV71 3D protein was found to be antiviral [88,89]. Interestingly, the pathogenesis of EV71 is determined by GSDME-mediated pyroptosis. EV71 induces caspase-3-dependent cleavage of GSDME, resulting in pyroptotic cell death. GSDME deficiency causes reduction of pathological symptoms and prolonged survival of EV71-infected mice [90].

### Ferroptosis

There is limited literature on RV infection and ferroptosis, although RV was found to induce ROS leading to damage to nasal epithelial barrier function [91].

Early RV infection is a risk factor for subsequent asthma, and the infection causes nearly two thirds of childhood asthma exacerbations [92]. The genetic variants of *ORMDL3* and *GSDMB* on human chromosome 17q21 are highly associated with asthma. Individuals homozygous for risk alleles at the *ORMDL3*-associated single nucleotide polymorphisms (SNPs) had a greater than twofold difference in the association between early viral infection and asthma [93,94]. *ORMDL3* has been associated with ferroptosis in some disease states and could have multiple pathways to influence RV infection [95]. SNPs regulating expression of GSDMB (implicated in pyroptosis) in airway epithelial cells play a major role in childhood-onset asthma [96].

Virus-induced ferroptosis may be a double-edged sword. Firstly, ferroptosis can cause cell death, limiting virus replication and spread. However, ferroptosis induces other chemokines and cytokines that can promote viral replication and reinfection. Viral activities such as viral gene expression, host–virus-triggered signalling, and virus-physiological stress can destroy organelles of the host. The destruction of cellular organelles that house abundant iron-containing or iron-requiring proteins such as lysosomes and mitochondria releases iron into the cytosol, inducing ferroptosis [97].

## Programmed cell death in asthma

Several studies have shown over the years that the death of eosinophils and epithelial cells in asthmatic airways plays a regulatory role in severe asthma [98–102], and these are summarised in Figure 3. Higher levels of LDH, a marker for cell death, were detected in serum of asthmatic compared with non-asthmatic subjects [103]. The present minireview focuses on PCD in eosinophilic asthma only, as there is limited literature on neutrophilic asthma.

**Figure 3:**
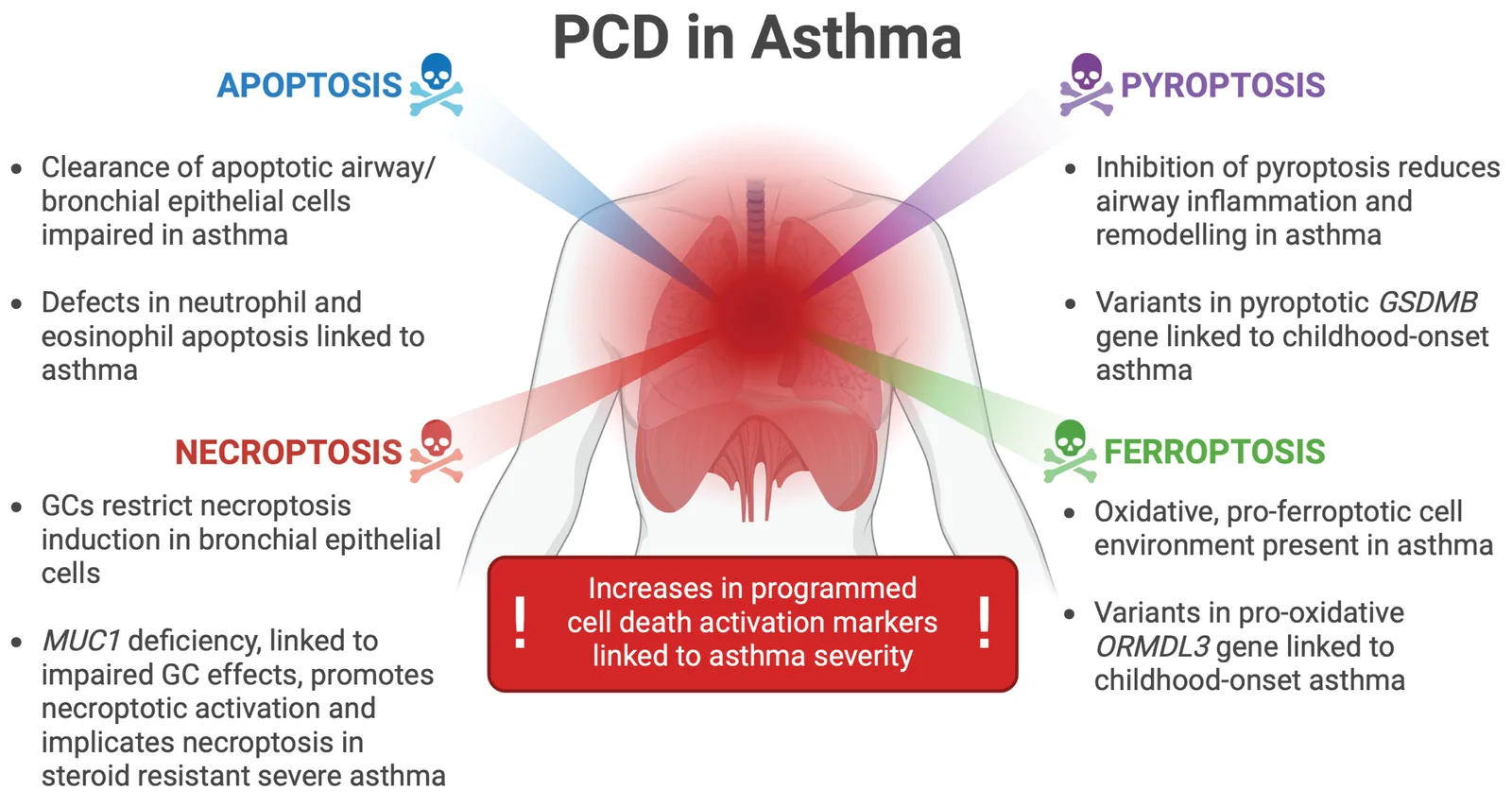
Overview of PCD in asthma Dysregulation of PCD pathways can contribute to the severity of asthma. Defective activation of apoptotic, necroptotic, pyroptotic, and ferroptotic signalling pathways contributes to asthma exacerbations. Increases in activation markers from all PCD pathways are associated with asthma severity.

### Apoptosis

While not fully understood, apoptosis has been shown to contribute to the pathogenesis of asthma, with several different cell types, including airway epithelial cells (AEC), eosinophils, and neutrophils, showing evidence of apoptotic cell death.

Several studies have found evidence of apoptotic AECs in asthmatic patients [104–106]. Significantly more epithelial cell apoptosis is observed in the airway of patients with severe asthma compared with mild asthmatics [107]. Creola bodies, clusters of uncleared apoptotic BECs, are found in the sputum of asthma patients [108]. Interestingly, Creola bodies may be a positive predictor of future asthmatic disease in children [109,110]. Clinical observations were corroborated in experimental *in vivo* mouse models of allergic airway disease [111–113].

Eosinophilic inflammation of the airway is a common observation in asthma. Defects in eosinophil apoptosis are thought to play a role in enabling the persistence of these cells in the asthmatic airways; preventing the resolution of eosinophilic inflammation and inducing apoptosis in eosinophils is a possible treatment strategy (reviewed in [114]). Neutrophilic inflammation is observed during asthma exacerbations and, importantly, in subgroups of patients with severe asthma that is often steroid refractory. Like eosinophils, neutrophils have been shown to have delayed apoptosis in asthma, with numbers of apoptotic neutrophils decreasing with increasing severity of disease [115–117] (Table S1).

### Necroptosis

Inhaled glucocorticoids (GCs) are the mainstay of asthma management. However, a small population of patients with severe asthma are resistant to even high doses of GCs [118]. In these patients, the increased TNF-α levels in the airway inhibit GC signalling [119]. Mucin 1 (MUC1) has important anti-inflammatory roles in the airways, and its expression is decreased in patients with severe asthma [120]. MUC1 deficiency leads to reduced anti-inflammatory effects of dexamethasone [120], is implicated in modulation of apoptosis, and augments TNF-α-induced necroptosis in human BECs [121]. Interestingly, the anti-necroptotic effect of GCs was impaired on MUC1 down-regulation [122], suggesting a potential mechanism for steroid resistance in severe asthma. Data from asthmatic patients and mouse models of asthma showed the involvement of necroptosis in asthma, with eosinophils being the main cell type impacted [103]. Elevated levels of phosphorylated MLKL and RIPK3 [123] are found in the serum of asthmatic patients. TNF-like cytokine (TL) 1A, an important regulator of Th2 inflammation in asthma, induced necroptosis through its receptor, DR 3, in the context of caspase inhibition [123]. This effect was more obvious in a mouse model of asthma than in cell culture. Importantly, TL1A-induced pathology in the animal model was reversed by MLKL knockout.

Together, the limited literature suggests a role for necroptosis in asthma overall and in the steroid-resistant severe asthma that accounts for 50%–80% of health care costs associated with asthma [124].

### Pyroptosis

Genetic variants at the *ORMDL3/GSDMB* locus are strongly and consistently associated with childhood-onset disease [125,126]. GSDMB is highly expressed in lung bronchial epithelium in human asthma and associated with asthma severity and asthma exacerbations [127,128]. Importantly, inhibition of pyroptosis signalling pathways reduces airway inflammation [129] and remodelling in animal models of asthma [130,131]. Other biomarkers of pyroptosis, such as NLRP3, IL-18, IL-1β, and caspase-1, were also found elevated in airway biopsies and/or serum of patients with pulmonary diseases and associated with disease severity [132].

### Ferroptosis

There is strong evidence that an imbalance between the reducing and oxidizing systems favouring an oxidative state is present in asthma [133]. Oxidative stress can cause airway hyperresponsiveness and airway inflammation [134]. Multiple biomarkers of lipid peroxidation have been identified in patients with asthma, suggesting ferroptosis is involved in the pathophysiology of asthma [135]. Increased iron levels in the lungs are associated with worsened asthma symptoms and reduced lung function [136].

## RV-induced programmed cell death in asthma

Most asthma exacerbations are associated with viral respiratory infections, and viral infections are closely associated with subsequent wheezing illnesses in children. The major viruses that trigger asthma are RV, respiratory syncytial virus, influenza virus, and severe acute respiratory syndrome coronavirus 2. Approximately 60% of asthma exacerbations have been linked with RV infections [137,138], and RV-C infections are responsible for most asthma attacks in children that result in hospitalisation [139].

There is limited literature on PCD in RV-induced asthma exacerbations, and none in the context of neutrophilic asthma. Given that PCD pathways are implicated in asthma and modulated in RV infection, it leads us to suppose that some of the clinical outcomes of asthma exacerbations may be due to enhanced PCD dysfunction/dysregulation (Figure 4). Indeed, a study in adults presenting to the emergency department with acute asthma found significantly higher sputum LDH activity in patient groups with infection-related acute asthma compared with those with non-infective acute asthma [71].

**Figure 4:**
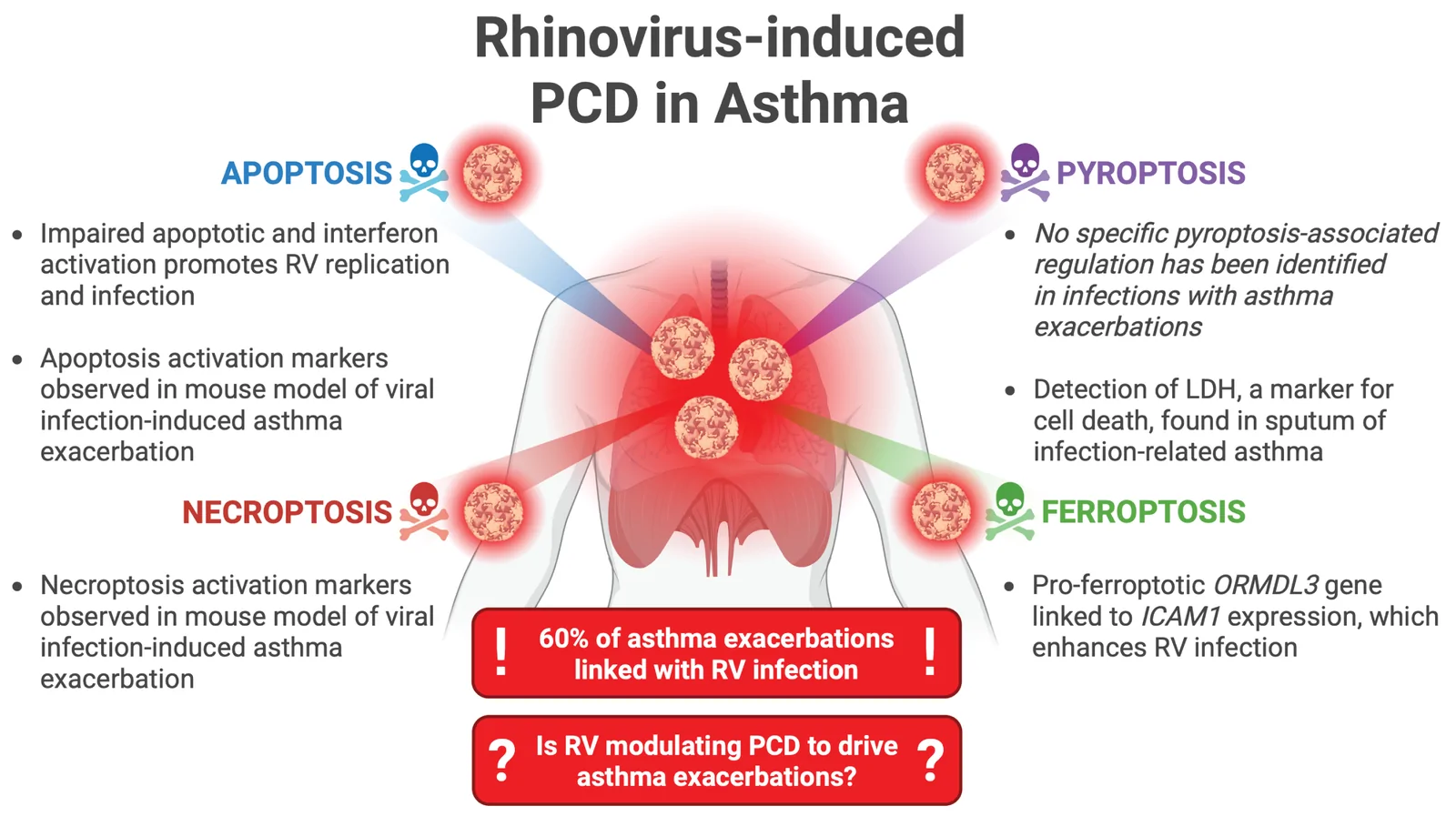
RV-induced PCD in asthma exacerbations The activation of PCD pathways is associated with increased asthma exacerbations during RV infection. The modulation of PCD pathways by RV during infection corresponds with markers of activation of apoptosis, necroptosis, ferroptosis, and potentially pyroptosis. Modulation of PCD pathways by RV may be driving asthma exacerbations during RV infection.

Primary cells from some asthmatic patient airways, particularly those with poorly controlled disease, are deficient in IFNβ and hence unable to restrict RV infection [66,140–143]. BECs from asthmatic airways have an abnormal innate response following RV infection, with impaired type 1 IFN production and apoptosis, increased virus replication, and cell lysis compared with healthy controls [66,71]. Exogenous IFNβ induced apoptosis and reduced virus production to levels seen in non-asthmatic BECs [66]. Similarly, AECs from asthmatic children supported higher proliferation of RV than AECs from non-asthmatic children. The reduced apoptosis and IFNβ response and increased inflammatory cytokines were rescued by exogenous IFNβ [144]. In a mouse model of asthma exacerbation based on house dust mite (HDM) sensitisation followed by dsRNA (representing virus infection), significant LDH release and increased markers of necroptosis (increased pMLKL) and apoptosis (cleaved caspase-3) were observed [145]. Interestingly, mice deficient in IFNβ had higher levels of LDH, pMLKL, and IL-1β at exacerbation (HDM:dsRNA) compared with wild-type mice. PCD pathways in mice are not the same as in humans; however, the findings of the present study are suggestive of an additive PCD effect of RV infection in asthma.

RV infection causes increased expression of caspase-1 in BECs in asthma [146], maybe through modulation of pyroptosis. GSDMB is overexpressed upon RV infection in asthma, associated with activation of the interferon pathway [123]. ORMDL3 influences expression of intercellular adhesion molecule-1 [147], a major RV receptor. Together with its role in lipid mechanisms and cell stress, this makes ORMDL3 a good candidate to regulate ferroptosis in asthma exacerbations caused by RV infection.

Ferroptosis, apoptosis, necroptosis, and pyroptosis, together, can eliminate the virus in cells, while on the other hand, can support virus replication. Understanding PCD pathways induced by RV will help us to find new therapeutics to treat asthma exacerbations in future.

## Hypotheses and research direction

We hypothesise that respiratory virus-induced dysregulation of PCD pathways—including apoptosis, pyroptosis, necroptosis, and ferroptosis—plays divergent, context-dependent roles in asthma pathogenesis. These pathways can compromise epithelial integrity, promote aberrant immune responses, impair viral clearance, and delay tissue repair, contributing to chronic inflammation and airway remodeling.

Emerging evidence supports the pathological role of PCD in asthma. Inhibitors targeting cell death mediators—NLRP3, caspase-1, GSDMs, and RIP kinases—have shown therapeutic potential in reducing inflammation in preclinical models. Repurposed drugs like disulfiram (GSDMD inhibitor) and anakinra (IL-1 receptor antagonist) that inhibit pyroptosis, as well as necroptosis inhibitors, antioxidants, and iron modulators, offer translational promise. Biomarker-guided patient stratification is essential to tailor treatment strategies and personalised approaches (e.g., IL-1β for pyroptosis, 4-HNE for ferroptosis, and pMLKL for necroptosis) that could optimize treatment in steroid-refractory cases. The preferred therapeutic direction depends on the PCD pathway, the affected cell type, and the stage of infection. Furthermore, inhalable formulations incorporating nanoparticle-based delivery systems warrant exploration. Such systems may facilitate targeted drug delivery to the airway epithelium while minimizing systemic side effects, thereby enhancing safety and efficacy.

PCD-targeted therapies require careful timing, e.g., early apoptosis inhibition may impair viral clearance, while broad apoptosis induction may worsen epithelial damage. Similarly, indiscriminate antioxidant or iron-chelating treatment may affect normal cellular functions. PCD-directed treatment should be temporally restricted, locally delivered where possible, guided by biomarkers, and potentially combined with antivirals or interferon-restoring therapies.

Future research should focus on developing human-relevant models (e.g., iPSC-derived organoids, lung-on-a-chip) to dissect virus-specific PCD mechanisms. Single-cell and spatial omics can uncover cell-type-specific responses, and longitudinal studies correlating PCD biomarkers with clinical outcomes may enable molecular endotyping. Ultimately, an integrated strategy combining modeling, biomarker validation, and targeted therapy is essential to translate these findings into personalised treatment for asthma.

## Perspectives

- Importance of the field: RV-induced exacerbations continue to account for the majority of virus-related asthma exacerbations, with estimated healthcare costs in hundreds of billions of dollars. There are no effective treatments for RV-induced asthma exacerbations. By examining the cellular and molecular pathways of PCD in response to RV infection in asthma, the present review aims to direct future research for therapeutic development.
- Summary of current thinking: Several types of PCD have been implicated in asthma, including apoptosis, necroptosis, pyroptosis, and ferroptosis. RV infection of epithelial cells triggers an inflammatory response, which can provide the necessary pro-inflammatory chemokines and cytokines to initiate PCD. At the same time, RV exploits PCD to aid its replication and release, while modulating them probably to limit immune response. The RV modulation of PCD, overlaid on already dysregulated pathways, could contribute to the observed asthma exacerbation.
- Future directions: Repurposed drugs that target PCD show translational promise. To translate these insights into clinical benefit, an integrated strategy is needed—including advanced *in vitro* modelling, mechanistic *in vivo* or clinical validation, biomarker-guided stratification, and new modality with innovative drug delivery systems. Understanding cell-type-specific responses will support biomarker-guided patient stratification that is critical for personalised treatment strategies, especially for steroid-refractory cases.

## Open Access

Open access for this article was enabled by the participation of Imperial College London in an all-inclusive Read & Publish agreement with Portland Press and the Biochemical Society under an agreement with JISC.

## Abbreviations

AEC: airway epithelial cells
BEC: bronchial epithelial cells
CT: C-terminal
DAMP: damage-associated molecular pattern
EV: enterovirus
GCs: glucocorticoids
GSDM: gasdermin
GSDMB: Gasdermin B
GPX: glutathione peroxidase
HDM: house dust mite
IFN: interferon
IL: interleukin
LDH: lactate dehydrogenase
LPS: lipopolysaccharide
MLKL: mixed lineage kinase domain-like protein
MUC1: Mucin 1
NLRP: NLR family pyrin domain-containing
NT: N-terminal
PCD: programmed cell death
RIPK3: receptor-interacting serine/threonine-protein kinase 3
ROS: reactive oxygen species
RV: rhinovirus
SNPs: single nucleotide polymorphisms
TL: TNF-like cytokine
TNF: tumour necrosis factor
ZBP: Z-DNA binding protein
